# Artificial intelligence in the radiomic analysis of glioblastomas: A review, taxonomy, and perspective

**DOI:** 10.3389/fonc.2022.924245

**Published:** 2022-08-02

**Authors:** Ming Zhu, Sijia Li, Yu Kuang, Virginia B. Hill, Amy B. Heimberger, Lijie Zhai, Shengjie Zhai

**Affiliations:** ^1^ Department of Electrical and Computer Engineering, University of Nevada Las Vegas, Las Vegas, NV, United States; ^2^ Kirk Kerkorian School of Medicine, University of Nevada Las Vegas, Las Vegas, NV, United States; ^3^ Medical Physics Program, Department of Health Physics, University of Nevada Las Vegas, Las Vegas, NV, United States; ^4^ Department of Radiology, Feinberg School of Medicine, Northwestern University, Chicago, IL, United States; ^5^ Department of Neurological Surgery, Feinberg School of Medicine, Northwestern University, Chicago, IL, United States; ^6^ Malnati Brain Tumor Institute of the Lurie Comprehensive Cancer Center, Feinberg School of Medicine, Northwestern University, Chicago, IL, United States

**Keywords:** artificial intelligence, machine learning, brain tumor, immunotherapy, radiomics, tumor classification, survival prediction, radiogenomics

## Abstract

Radiological imaging techniques, including magnetic resonance imaging (MRI) and positron emission tomography (PET), are the standard-of-care non-invasive diagnostic approaches widely applied in neuro-oncology. Unfortunately, accurate interpretation of radiological imaging data is constantly challenged by the indistinguishable radiological image features shared by different pathological changes associated with tumor progression and/or various therapeutic interventions. In recent years, machine learning (ML)-based artificial intelligence (AI) technology has been widely applied in medical image processing and bioinformatics due to its advantages in implicit image feature extraction and integrative data analysis. Despite its recent rapid development, ML technology still faces many hurdles for its broader applications in neuro-oncological radiomic analysis, such as lack of large accessible standardized real patient radiomic brain tumor data of all kinds and reliable predictions on tumor response upon various treatments. Therefore, understanding ML-based AI technologies is critically important to help us address the skyrocketing demands of neuro-oncology clinical deployments. Here, we provide an overview on the latest advancements in ML techniques for brain tumor radiomic analysis, emphasizing proprietary and public dataset preparation and state-of-the-art ML models for brain tumor diagnosis, classifications (e.g., primary and secondary tumors), discriminations between treatment effects (pseudoprogression, radiation necrosis) and true progression, survival prediction, inflammation, and identification of brain tumor biomarkers. We also compare the key features of ML models in the realm of neuroradiology with ML models employed in other medical imaging fields and discuss open research challenges and directions for future work in this nascent precision medicine area.

## Introduction

Glioblastoma (GBM, WHO grade 4 glioma, IDH-wildtype) is the most aggressive primary brain tumor in adults with a dismal median overall survival (OS) of only 12 to 18 months and a 5-year OS rate of 6.8% (1, 2). Approximately 13,000 GBM cases are diagnosed in the United States each year, with an incidence rate of 3.2 per 100,000 members of the population (3, 4). Despite standard-of-care therapy including aggressive surgical resection followed by radiation therapy and chemotherapy, more than 90% of glioblastomas recur (4). To date, magnetic resonance imaging (MRI) remains the standard approach in the diagnosis, prognosis, and therapeutic monitoring of GBM patients because it is non-invasive, accessible, and cost efficient. However, interpretation of radiological imaging data can be subjective, challenging, and time-consuming, mainly because histologic findings are often radiologically occult. For example, therapy-induced treatment effect (i.e., pseudoprogression (PsP) or radiation necrosis) and true tumor progression manifest with identical MRI appearances, and differentiation between these entities remains an unsolved conundrum in current neuro-radio-oncology, particularly with novel therapies such as immune checkpoint inhibitors (5).

Radiomics (6, 7) in neuro-oncology seeks to improve the understanding of the biology and effects of treatment on the imaging appearance of brain tumors. Radiomics can promote precision medicine by extracting quantitative features from clinical imaging arrays and using methods from the field of artificial intelligence (AI) to make the radiological diagnosis more objective, accurate, and automatic. Rather than designing hard-coded step-by-step algorithms based on prior knowledge in biology or medicine, or design specific “learning” approaches to mimic human cognitive functions, machine learning (ML) as a subfield of AI can create a computational model and train it with a number of datasets to statistically solve problems without being explicitly programmed (8). Generally, ML includes supervised learning, unsupervised learning, and reinforcement learning. Supervised learning trains an ML model to predict a target variable from a set of predictive variables (i.e., data samples) with the help of labels/annotations (i.e., ground truth of the target variables) and the loss function, also known as cost function, which is a computational difference between predicted target variable values and the label/annotation values (9). It should be noted that, although labeling and annotation share the same meaning in ML, they slightly differ in neuro-oncology radiomic analysis. In the context of this manuscript, labeling is related with classification problems (e.g., the ground truth of tissue is histological, including different classes of brain tumors, treatment effect versus tumor growth, and others) whereas annotation refers to segmentation problems (partitioning an image into multiple regions/objects, such as enhancing tumor, necrosis, and unenhancing tumor and edema). Unsupervised learning infers the inherent structure from the input data without labels/annotations (10). In reinforcement learning, intelligent agents learn to take actions in an environment in order to maximize the notion of cumulative reward (11). Currently, most AI techniques applied in brain tumor radiomic studies belong to the supervised ML, for both classifications and segmentations. Unsupervised ML is mainly employed for image segmentations while reinforcement learning has not been explored in this area. Therefore, in this paper, we mainly focus on the supervised ML techniques for most GBM radiomic analyses. Thanks to the rapid development of radiology and computational hardware, researchers can now take advantage of many radiological data to train various ML models, such as decision trees (DTs), logistic regression, artificial neural networks (ANNs), support vector machines (SVMs), and *k*-nearest neighbors (*k*-NN) for brain tumor radiomic analysis. The different techniques applied in AI (mainly ML algorithms) are the technical core for the analysis of large amounts of multidimensional radiologic and clinical data (12), which directly determine the quality of radiomic analysis results.

**Figure 1:**
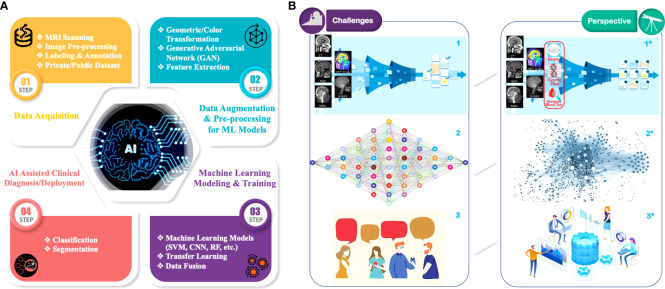
AI/ML in GBM radiomic analysis: **(A)** Overall workflow of AI-assisted GBM analysis: 1) *Data Acquisition*. Raw radiological image data are acquired by MRI scanning of GBM patients. Images are collected into public or private data sets. Before analysis, images are preprocessed (e.g., data cleaning, co-registration, normalization) and standardized (e.g., format, resolutions, voxel sizes). Then, radiologists annotate the images, color-coding different parts of the tumor habitat. 2) *Data Augmentation and Preprocessing for ML Models*. Imaging dataset and its annotations from step 1 are further “augmented” *via* geometric transformations, photometric transformation, and/or synthetic data (e.g., GAN) to improve the data generalizability, followed by the optional preprocessing for ML modeling, a process that includes feature extraction to filter out “useless” data and extract explicit features (e.g., biological and/or geometry) in the images. 3) *ML modeling and training*. Augmented and preprocessed data are fed into various ML models (e.g., SVM, RF, CNN) for GBM radiomic analysis training and validation. Advanced techniques such as transfer learning and multimodal data fusion (e.g., clinical and genomic data) can be employed to improve the training accuracy as well as generality. 4) *AI-Assisted Clinical Diagnosis/Deployment*. Predictions from the ML models for various medical demands, such as differential diagnosis and survival estimation. **(B)** Current major challenges (left panel: 1, 2, 3) and perspectives for corresponding solutions (right panel: 1*, 2*, 3*) in AI/ML-assisted GBM radiomic analysis: 1→1* Current GBM radiological image datasets are limited in low numbers, insufficient annotations, and poor organization. Enrichment and standardization of current GBM radiological datasets are urgently needed, while incorporation of clinical and/or genomic data (red circle) can further enhance the performance of ML prediction models; 2→2* develop more comprehensive ML models to further improve the prediction accuracy and address the relatively low generalizability of current models; 3→3* further strengthen collaborations among clinicians, biomedical researchers, and computer scientists to overcome the lack of efficient communications between these parties for the highly multidisciplinary research.

In the past decade, ML has been widely exploited in many data-driven applications, e.g., imaging and computer vision (13), bioinformatics (14), online advertising (15), and natural language processing (16). The dataflow of a general supervised ML-based GBM radiomic analysis can be divided into four steps as shown **Figure 1A**: 1) *Data Acquisition*. MRIs are performed on patients with a brain tumor. These raw MRI data are further preprocessed (e.g., data cleaning, co-registration, bias correction, normalization), and then they are labeled/annotated by radiologists to define the regions for the ML training and validation process. The labeled/annotated imaging data are deposited into customized/private datasets that are owned and maintained by medical research institutions. Some imaging data are also uploaded into public datasets for the purpose of open access to all researchers. AI-assisted radiomic analysis can acquire imaging data from both types of datasets. It is of note, however, that private datasets usually contain a fairly large amount of raw data, hundreds of samples for each institution if applicable, whereas public datasets usually contain limited amount of less well-labeled/annotated, non-standardized imaging data (17); 2) *Data Augmentation and Preprocessing for ML Models*. The acquired data and its labels/annotations are usually first subject to augmentation, in which image data are processed in pair-wise format (i.e., each pair contains a data sample and the corresponding labels/annotations) to increase the sample variety, hence improving the generality of the data. Multiple approaches are utilized in augmentation, such as geometric transformation, color augmentation, and synthesis of similar-appearing imaging data. Then, the augmented imaging data can be preprocessed (e.g., through feature extraction) to simplify and/or to improve the effectiveness and efficiency of the subsequent ML training process (18); 3) *ML Model Training and Validation*: The augmented and preprocessed data are subsequently fed to ML models to train the model parameters in order to minimize the “cost function,” while the implicit data feature will be extracted statistically. The ML models are also validated during the training process to prevent overfitting, which is when the trained ANN model only predicts accurately to the training dataset but loses the generalizability to new samples (19); 4) *AI-Assisted Clinical Diagnosis/Deployment*. Once the trained models meet the accuracy requirement, they can be deployed to the application to perform predictions such as classification and segmentation. As aforementioned, no biologic hypothesis or knowledge is required to build an ML model. However, inclusion of this information and/or other forms of data (e.g., clinical data, genomic data) may help with the overall ML model performance by reducing the data size or improving the data quality during the preprocessing step.

Traditional ML methods such as SVMs (20–22) and random forests (RFs) (23–26), an ensemble combination of decision trees, are commonly used for pattern classification in tumor studies. Recently, ANN, especially convolutional neural network (CNN)-based deep learning (DL), is gaining popularity because of its improved scalability and the capability of exploiting deep layers to extract implicit local and global features in neuro-oncology images. They can achieve state-of-the-art performance in object detection and tracking (27, 28), image classification, and semantic segmentation (25, 29–32). In ML, each “neuron” is referring to a computational unit in the ANN rather than a biological neural. With more complicated ML models and structures (e.g., more neuron network layers, more neurons in each layer) and a larger number of parameters introduced into the neural network, the training process intends to extract more features but may suffer extensive computational performance degradation and overfitting of the trained model. Current state-of-the-art ML models can achieve an accuracy as high as 0.97 (i.e., 97%) in brain tumor radiomic analysis (33). However, these results are based on a limited number of datasets and from retrospective studies, which may still not be generalizable for patients from different geographic locations. Therefore, current clinical brain tumor radiomic analysis cannot entirely rely on the ML-based techniques and still needs manual verification. In summary, existing ML techniques can only partially fulfill the need for automatic detection and analysis of GBM characteristics for both clinical and preclinical studies (34–37).

In addition to the ML techniques, the quality of radiologic images that are used for ML training dramatically affects the outcomes of radiomic analysis. Radiological images can be acquired from different imaging modalities, such as MRI, computed tomography (CT), and positron emission tomography (PET). Among these, MRI image data are currently employed as an essential data type in radiomic neuro-oncology applications because 1) they provide exquisite detail of brain, spinal cord, and vascular anatomy through excellent tissue contrast in any imaging plane; 2) different MRI sequences are able to capture key components of tumor biology with high sensitivity, such as blood–brain barrier breakdown, necrosis, edema, non-enhancing tumor infiltration, blood flow, and cellular density, and can distinguish tumoral sub-compartments that are likely to reflect local cellular phenotypes and genotypes; and 3) they can non-invasively and non-destructively interrogate tumors repeatedly to assess response to treatment and thus they can be integrated into therapeutic strategies. Understanding these image-based features is critical as they not only represent a key data resource in radiomic analysis (6) but also help improve the accuracy and other performance criteria of ML models.

In this review, we provide an overview of the latest advancements and in-depth discussions on the most urgent and challenging questions of AI-assisted GBM radiomic analysis. Given the exponential increase of AI-based radiomic studies led by researchers from various backgrounds, such as oncology, radiology, computer science, and engineering, our review article briefly explains the key concepts of ML techniques instead of delving into the technical details. This article is structured with emphases on the deployments of various ML techniques in meeting specific GBM radiomic clinical needs, e.g., differentiating GBM from other brain tumors or non-tumors, predicting overall survival (OS), and correlating with other biomarkers. First, as ML technology in radiomics is radiological imaging data-driven, we start with the discussion on imaging data preparations that are commonly employed in current GBM radiomic analyses. We briefly introduce the acquisition pipeline for private or customized imaging datasets and summarize public radiologic datasets that are currently available for researchers to train their ML models for various applications. We also describe general methods for data augmentation and preprocessing for ML models, which are critical for training, validation, and testing of ML algorithms. Next, we overview the ML techniques that have been employed in radiomic analysis for GBM diagnosis and treatment. Advantages and limitations of existing ML models including both algorithms and architectures are discussed in the context of various GBM-associated medical applications. Finally, we bring up our perspectives on the strategies for overcoming challenges regarding AI/ML applications in GBM radiomic analysis, including 1) the most challenging issues affecting the generalizability and accuracy of AI-assisted radiomic GBM analysis; 2) promising strategies to enhance performance of AI models in GBM radiomic analysis; 3) outlook on the collaborative teamwork between computer scientists, engineers, physicians, and biomedical researchers. By elaborating current research developments and challenges in the state-of-the-art ML-assisted GBM analysis, we hope to inspire researchers from different fields for the development of the next generation of AI-assisted radiomic tools that can significantly improve early detection, treatment efficacy, and life quality of patients with GBM.

## Radiomic data preparation

As ML is an intensive data-driven algorithm/process, the quality of the training data can significantly influence the trainable parameters in ML models, hence affecting the accuracy and generalizability of the network outcomes (38). Thus, it is essential to review the key components of data preparation in ML-based GBM radiomic analysis, including radiological imaging data resources, the image acquisition pipeline, imaging datasets (private/customized datasets vs. publicly accessible datasets), data augmentation, and preprocessing techniques for the subsequent ML model training.

### Radiomic image resources

MRI is the most frequently used radiological modality for brain tumor imaging. MRI provides better contrast resolution than CT, with better tissue characterization. It can also detect blood vessels, vascular malformations, and demyelinating disease (39). It does not involve X-rays or the use of ionizing radiopharmaceuticals, either. Therefore, MRI is particularly suitable to image gliomas. Yet, MRI may be perceived as less comfortable by patients (e.g., overweight or fear of enclosed spaces) and cannot be performed if the patient has ferromagnetic implants. In addition, MRI does not show ossified or calcified structures as well as CT (such as the calvarium) and therefore may not show the effects of tumors on the calvarium in comparison to CT (39, 40).

For GBM MRI, T1-weighted (T1), T1-contrast-enhanced (T1c or T1-ce), T2-weighted (T2), and fluid-attenuated inversion recovery (FLAIR) are the most commonly used MRI sequences, because they can provide different yet complementary information in characterizing tissue such as gray matter, white matter, fat, blood, fluid, and lesions (41, 42). MRI is based on radiofrequency pulses within a magnetic field in which time of repetition (TR) and time of echo (TE) are calculated. T1 and T1-ce are produced through short TR and TE times; T2 is produced by larger TR and TE times; and FLAIR is produced through very large TR and TE times. In addition, diffusion-weighted imaging (DWI) can detect the restriction of random movements of water molecules that makes DWI extremely sensitive to detect acute stroke and increased cellularity as in GBMs, lymphoma, and metastases (43); magnetic resonance angiography (MRA) and venography (MRV) can generate pictures of the arteries and veins to evaluate for stenosis or aneurysms; magnetic resonance spectroscopy (MRS) is used to measure the levels of different metabolites and biochemical changes in the brain (44), providing information on tumor metabolism (45, 46); perfusion-weighted imaging (PWI) shows the perfusion of tissues by blood, such as the cerebral blood volume of a tumor relative to normal-appearing white matter of the brain (47); and functional MRI (fMRI) detects the increase in blood oxygen level when blood flow increases to a brain area involved in the performance of an assigned task (e.g., finger tapping, lip pursing, thinking of words, thinking of answers to questions after hearing a story) (48) and depicts where eloquent brain areas are in relation to the tumor as the surgeon or radiation oncologist plans a surgical approach to biopsy or resection or radiation therapy. The novel amide proton transfer (APT) imaging can detect amide protons of endogenous mobile proteins and peptides in tissue based on chemical exchange–dependent saturation transfer (CEST) MRI (49, 50).

Another useful radiological technology is the PET scan, which takes advantage of a slightly radioactive substance (e.g., C-11 methionine (MET), F-18 fluorothymidine (FLT), F-18 fluoroethyl-L-tyrosine (FET)) that functionally is preferentially taken up by tumor cells (51). PET is especially helpful for fast-growing (high-grade) tumors and for distinguishing between tumors and non-tumor (e.g., scar, inflammation) tissue (52, 53). Therefore, the combined use of MRI and PET can provide complementary information to achieve more accurate brain tumor diagnosis (54).

### Radiological image acquisition pipeline

The image acquisition pipelines remain very similar between radiological scanners (50, 55). Subjects undergoing MRI (i.e., patients and control subjects) are usually examined on a clinical 1.5T or 3T scanner with a multichannel receive-only head coil array under various scanning parameters (e.g., TR, TE, field of view, matrix/voxel size). A sequence of 2D and 3D radiologic images (e.g., various MRI, CT, PET) is obtained. These images are cleaned, normalized, and co-registered (i.e., image preprocessing, **Figure 1A**). It is worth noting that different image intensity normalization schemes may influence not only the registration (56) and segmentation process (57, 58) but also the implicit texture features hidden in the different modal MR images and thus affect the subsequent feature selection and ML training outcomes for various GBM classification applications (59). Shinohara et al. (60) introduced a set of seven statistical principles of image normalization (SPIN). In addition to the common mean-maximum (or standard deviation) normalizations (59) and histogram-based normalizations (57, 61, 62), Shinohara et al. (60) also proposed a hybrid multimodal normalization method to match the natural balance of tissue intensities with physical interpretation. On the other hand, some data may be standardized (e.g., voxel sizes, resolution) while others may not. Then, the images are labeled and annotated into various categories of tissues and/or lesions by experienced radiologists using a variety of software (e.g., ITK-SNAP (63), 3D Slicer (64)) to produce a labeled/annotated imaging data set (17).

### Major public datasets

Since not all ML researchers can directly access private/customized high-quality labeled/annotated brain tumor datasets, which are usually owned and protected by medical institutions, public datasets are essential and provide an equal platform to these researchers to train and compare the outcomes of their ML models. In neuro-oncology, one of the most commonly used public online image datasets is from the Brain Tumor Segmentation (BraTS) challenges, organized by the Medical Image Computing and Computer Assisted Interventions (MICCAI) and other professional organizations (34, 65–69) since 2012. As of March 2022, the latest BraTS 2021 consists of a total of 2,040 brain tumor cases/patients, and it is divided into three subsets: training (1,251 cases), validation (219 cases), and testing (570 cases). Only training and validation subsets are open to the public research access, and these two subsets include a set of multimodal 3D MRI scans (i.e., T1, T1c, T2, FLAIR) for each case. The training dataset also includes a 3D annotation model (i.e., GD-enhancing tumor, peritumoral edema/non-enhancing infiltrative tumor, necrotic tumor core (NCR), and normal) for each case (17). In addition, BraTS 2021 includes O6-methylguanine-DNA-methyltransferase (MGMT) biomarkers for 585 patients (out of a total of 1,251 cases) for the training datasets. It should be noted that, unlike BraTS 2020, BraTS 2021 does not include survival information any longer. BraTS 2020 included the survival information for 265 patients (out of a total of 460 cases) in the training and validation datasets. Other widely used datasets include The Cancer Imaging Archive (TCIA) (70) and The Whole Brain Atlas by Harvard Medical School (71). TCIA has a collection of 13 brain tumor sub-datasets, including the Ivy Glioblastoma Atlas Project (IvyGAP) (72), The Cancer Genome Atlas (TCGA)-GBM (73), GLIS-RT (74), and CPTAC-GBM (75). These sub-datasets mainly focus on high-grade glioma (HGG/GBM) and lower-grade glioma (LGG). Some of the data in TCIA are also included and standardized in the most recent BraTS 2021 dataset. The Harvard brain atlas consists of the radiology data (e.g., MRI, CT, PET) for about 40 subsets of normal brain and various brain disease states. However, none of the datasets in the TCIA or Harvard brain atlas are pixel-wisely annotated for the ML segmentation tasks. In addition, the data format for each patient in TCIA collection varies in terms of pulse sequences (e.g., T1, T1c, T2, FLAIR, PWI, DWI) and the resolution (i.e., with matrices varying from 128*128 to 896*896), even in the same sub-dataset (e.g., TCGA-GBM, IvyGAP). A summary of these datasets is depicted in **Table 1**.

**Table 1:** List of three major sources for radiomic neuro-oncology public datasets.

Dataset	Radiology data type	Data size	Image resolution
BraTS	3D MRI	2,040 subjects, including both HGG and LGG	240 * 240 * 155
TCIA	2D MRI, CT, axial slices	13 brain tumor sub-datasets, including IvyGAP (39 subjects), TCGA-GBM (262 subjects), and TCGA-LGG (199 subjects)	Varying from 128*128 to 896*896
Harvard Medical School: The Whole Brain Atlas	2D MRI, CT, PET, axial slices	8 subsets for brain tumors, and 30 other subsets for normal brains and other non-tumor brain diseases	256 * 256

### Data augmentation and preprocessing for the ML models

Data augmentation is a commonly used technique in ML for the purpose of promoting the accuracy and generalizability of the ML algorithms. Data augmentation can be attained by 1) adding slightly modified copies and/or 2) creating new synthetic data from already existing data. The former usually employs geometric transformation and photometric transformation including flipping, pixel-level augmentation, cropping, rotating, noise injection, and random erasing (76, 77), while the latter may make use of generative adversarial networks (GANs) (78, 79) to create new synthetic images that resemble the original dataset. It should be noted that GANs also belong to ML-based networks that require abundant training data to generate resembled data. Data augmentation acts as a regularizer and helps reduce class imbalance and overfitting (76), so as to improve both the accuracy and the generalizability of the ML outcomes.

Current GBM radiomic studies are often hindered by limited and unbalanced data samples; therefore, using ML models alone may not achieve statistically significant outcomes (80–82). In this regard, the preprocessing plays a vital role by enhancing and extracting some image features, especially the biological/medical meaningful ones in the regions of interests (ROIs), and/or filters out some “useless” image data from the datasets, before performing the ML training and analysis (18). General data preprocessing approaches for ML models include feature extraction and feature selection (18, 81). In GBM radiomic datasets, MRIs contain various features, such as image texture (23), local histograms (24), structure tensor eigenvalues (25), gray-level co-occurrence matrix (GLCM) (83), and local binary pattern (LBP) (41). Yet some of these features might be correlated in that the total number of effective features can be further reduced, by employing feature selection algorithms such as principal component analysis (PCA) (84), least absolute shrinkage and selection operator (LASSO) (85), linear discriminant analysis (LDA) (86), t-tests (87), analysis of variance (88), and information gain based methods (89), or based on certain evaluation criteria, such as probability of error (POE) and average correlation coefficient (ACC) (59, 90). Injecting feature extraction and selection can significantly help to reduce the computational complexity of ML models and speed up the training process and possibly improve the accuracy of ML models for brain tumor classification and segmentations (91, 92). Nevertheless, one should note that such preprocessing should be treated with care so that the ML model is not overfitted to particular features, which could lose generalizability to a different dataset.

### Discussion

We have illustrated various radiomic data sources and data preparation techniques that are commonly employed in ML-based GBM radiomic analysis. One prominent issue in current GBM radiomic data preparation lies in the lack of standardized image acquisition specifications (e.g., repetition time, echo time, voxel sizes, image resolutions) between different radiological equipment and medical institutions (i.e., multicenter multi-vendor, McMv, datasets), which may 1) bias the image data (e.g., intensity of pixels, actual voxel size); 2) require additional image data preparation (e.g., cropping, up/down-sampling) to train ML models with different datasets; and 3) impede the development and cross-validation of more general/robust and accurate ML models for McMv datasets. Although BraTS has made a huge effort and progress in standardizing radiology data for over 2,000 GBM patients/cases, it is not yet sufficient for various GBM analysis applications. The second important issue is that most existing datasets have limited types of brain tumors (e.g., GBM/HGG, metastasis, and LGG), while the Harvard Atlas is limited by the number of subjects/patients. Scarcity of brain tumor/disease types and lack of data impede the application of ML to accurately distinguish various brain tumors and diseases. Lastly, most datasets include only MRI data while only a few datasets consist of other modality radiology data such as CT and PET. Most of them do not include other biological information (e.g., survival time, histopathological data, biomarkers) either. There is also lack of longitudinal radiology data to show disease evolution for patients receiving various treatments. With more complementary data and medical/treatment history (including radiomic data) that help comprehensively describe the brain tumor/disease status, an improvement in the accuracy of radiomic analysis and prediction can be expected.

## Application of AI/ML in GBM diagnosis and therapeutic monitoring

The early brain tumor radiomic studies often relied on conventional radiomic feature-based ML methods that extract relatively explicit image texture features (e.g., shape, GLCM, LBP) to train traditional ML models such as SVMs (20–22) and RFs (23–26) in order to differentiate brain tumor versus non-tumors (or different types of brain tumors), predictions of overall survival, etc. Recently, by taking advantage of deep neural networks (DNNs) that include more neurons and layers to statistically recognize global, deep, and implicit imaging features, DL techniques can achieve state-of-the-art performance for automatic analysis of brain tumors on multimodality imaging and clinical data (32). Additionally, deep feature-based ML techniques build statistically/biologically meaningful models or utilize DNNs to extract deep implicit features from the radiology images and then apply traditional ML models for classifications (93, 94). Despite their differences, the above ML models all exploit prior biomedical and image features knowledge to 1) preprocess the radiological imaging data to extract imaging and/or biological meaningful features and 2) optimize the ML structure/algorithm for specific classification/segmentation tasks. Examples include the stacked denoising autoencoders (95) and the Convolutional Restricted Boltzman Machine (96). All these ML models have been applied in radiomic analysis to address unmet needs in GBM diagnosis, therapeutic monitoring, and/or prognosis (e.g., brain tumor classifications, survival predictions and biomarker identifications), while at this point, CNN-based DL enjoys the most generalizability and highest accuracy. Details are further discussed under the context of individual study case as follows.

### GBM diagnosis and classification

One of the major ML applications in GBM radiomic analysis is to facilitate the differentiation between GBM and other histopathological processes. More specifically, such applications mainly fall into three categories: 1) distinguish brain tumor from other non-cancerous pathologies; 2) distinguish GBM from other brain tumors; and 3) differentiate between true progression and treatment effect (PsP or radiation necrosis). We hereby provide an overview of a few of these classification problems in brain tumor diagnosis.

#### 1) Differentiating tumor from non-tumor

One of the most critical radiomic functions in brain tumors is to distinguish between malignant brain tumor and non-tumor pathologies, which include tumefactive demyelination, infection, inflammation (e.g., paraneoplastic syndromes and autoimmune disease), cortical dysplasia, and stroke. However, due to insufficient data available for ML training of each specific non-tumor type, existing studies mostly classify all data into two major categories: tumor (e.g., GBM/HGG, metastases, LGG) and non-tumor (i.e., control/normal and non-cancerous pathologies such as inflammation). Some studies tested their ML models on their own private data, while others took advantage of public datasets (e.g., BraTS, TCIA, Harvard brain atlas) or a combination of private and public data to expand the model’s generalizability. As discussed in Section 2.4, data preprocessing (e.g., filtering and feature extractions) are also often used to denoise and enhance the lesion region in the input MRI slides, with a hope to speed up the ML training process and improve the accuracy of distinguishing between tumors and non-tumors.

For those using public datasets, Ari et al. (97) proposed a three-phase extreme learning machine local receptive field (ELM-LRF) method for tumor classifications: removal of the noise using local and non-local methods, segmentation of benign or malignant tumor using ELM-LRF, and then use of a CNN classification. As a result, they achieved an effective classification accuracy of 0.97. Mohsen et al. (84) took advantage of a discrete wavelet transform (DWT) for feature extraction and principal component analysis (PCA) for reduction, together with a fuzzy C-means DNN to classify a dataset of 66 brain MRIs from Harvard Brain Atlas into four classes, i.e., normal, glioblastoma, brain sarcoma, and brain metastatic bronchogenic carcinoma tumors. An accuracy of 0.97 was achieved, and an area under the curve (AUC) approximated 0.984.

Alves et al. (83) quantified the gray-level pattern, pixel interrelationships, and the spectral properties of an image and achieved two fundamental features from an MRI sample, i.e., GLCM and gray-level run-length (GLRL). By combining this texture analysis with ML models (e.g., SVM, RF), they differentiated brain tumors from inflammatory lesions in their local MRI dataset and achieved a high accuracy of 0.83 and AUC of 0.906. Citak-Er et al. (93) applied a multiregional and multiparametric recursive feature elimination method, which was based on the Mann–Whitney ranking score, and then they employed the SVM-based multilayer perceptron (MLP) classification model to achieve a tumor detection accuracy of 0.93.

Amin et al. (41) mixed the public datasets and local datasets to differentiate tumors and non-tumors. They employed a Weiner filter to denoise and enhance the lesion region in the input MRI slides and used potential field (PF) clustering to identify the tumor region. Gabor wavelet transform (GWT) and LBP features were fused with various ML models (i.e., SVM, DT, *k*-NN, and naïve Bayes) to further improve the classification accuracy. The approach yielded an accuracy greater than 0.93 and an AUC of 0.96. Zhou et al. (98) treated holistic 3D MRI samples as sequences of 2D slices to extract some 3D features on brain tumors. They introduced a recursive structure, i.e., the long short-term memory (LSTM), to a deep CNN model (i.e., DenseNet) to handle such sequential data classification, and this DenseNet-LSTM model achieved an outstanding accuracy of 0.92 using the BraTS dataset (99).

Both Banerjee et al. (100) and Xu et al. (101) introduced transfer learning (TL) to improve the accuracy of the DNN-based ML classifier with non-brain-tumor images. They first pretrained the ML classifier with the large general image dataset, ImageNet (102), and then they fixed the pretrained parameters in the CNN hidden layers and fine-tuned the parameters in the output layers with neuro-oncology MRI images. Xu et al. (101) even embedded an SVM with the CNN to distinguish between GBM and LGG. By doing so, the two studies achieved classification accuracies of 0.97 and 0.975, respectively.

#### 2) Differentiating primary from secondary brain tumors

Secondary/metastatic brain tumors have as high as fivefold incidence of that of primary brain tumors and manifest a rapid growth, causing significant brain tissue damages. Patients typically present with multiple metastatic tumors throughout the brain (103). A traditional non-ML-based approach to distinguish multifocal GBM from metastases on *α*[^11^ *C*]-methyl-_L_-tryptophan (AMT)-PET images is to examine the tumoral standardized uptake values (SUVs), mean tumor/cortex SUV ratio, and tumor/cortex volume of distribution (VD)-ratio (104). Compared to GBM, metastases had lower values of all three parameters. However, this approach can only achieve an accuracy of 0.72.

Many studies implemented a combination of various feature extractions and regular ML models to find the best performance for their applications. Zacharaki et al. (86) introduced a Gabor texture filter with feature ranking to extract tumor features, and derived feature ranking scores, and then applied three ML models to distinguish GBM from metastases: SVM with recursive feature elimination (SVM-RFE), linear discriminant analysis (LDA, also known as Fisher linear discriminant) with Fisher’s discriminant rule (105), and *k*-NN. Among the three models, SVM-RFE achieved the highest mean accuracy and AUC of 0.91 and 0.936, respectively. Chen et al. (85) compared 30 diagnostic models that were built based on five feature selection models and six classification algorithms for distinguishing GBM and metastases. The five feature selection models included distance correlation, RF, least absolute shrinkage and selection operator (LASSO), eXtreme gradient boosting (XGBoost), and gradient boosting decision tree (GBDT), while the six classification algorithms included LDA, SVM, RF, *k*-NN, Gaussian naïve bayes (GaussianNB), and logistic regression (LR). The results showed that the combinational model of distance correlation and LR outperformed all other combinations in terms of testing accuracy (0.79) and AUC (0.80), although some other combinations achieved similar results as well.

Priya et al. (106) analyzed 60 GBM and 60 metastases cases with 12 regression or ML-based classifier models and four feature reduction/selection strategies—45 combinations in total. According to their results, the mean performance of various models was slightly better with FLAIR images than multiparametric sequences in terms of AUC, while the combination of full feature and LASSO achieved the highest AUC of 0.953, although full features with other models, such as ElasticNet (107) and RF, achieved similar results. de Causans et al. (55) trained on T1 MRI data with 71 GBM and 72 metastasis cases using 100 extracted features, based on the Image Biomarker Standardization Initiative (IBSI) (108). With these selected features, a total of 144 models combining nine feature scaling methods and 16 classifiers (regression and ML-based) were compared. All 144 classifiers of the 21 GBM and 16 metastases cases achieved a mean accuracy and AUC of 0.8 and 0.85, respectively.

#### 3) Differentiating GBM from primary central nervous system lymphoma

GBM and primary central nervous system lymphoma (PCNSL) are not only common intracranial malignancies but also often share similarities in radiological appearance. However, the management for each disease is quite different (109). Recently, multiple ML-based predictive analytics have arisen to help differentiate GBM from PCNSL radiologically with a relatively high sensitivity and specificity. Outcomes were assessed based on test characteristics such as accuracy, sensitivity, specificity, and AUC.

Due to the binary classification nature of distinguishing between GBM and PCNSL, SVM used to be the most commonly employed model for its computational simplicity (110–115). Other commonly used ML models such as *k*-NN and RF were also exploited and compared (114, 116). Even though the training datasets were relatively small, approximately 110 or fewer samples with about two-thirds of the entire dataset containing GBM and one-third of PCNSL, it turned out that most ML-based models were able to achieve an accuracy between 0.9 and 0.96, a sensitivity of 0.84 or higher, a specificity of 0.89 or higher, and AUC of 0.92 or higher. More notably, Kang et al. (114) and Suh et al. (116) even compared the prediction outcomes of their ML models with the prediction from human radiologists, and the results showed the superiority of ML models over human radiologists in all four criteria, especially in accuracy, sensitivity, and AUC. It is unknown whether the combination of the ML model and human radiologist read would have attained even higher accuracy, sensitivity, and AUC.

Recently, more sophisticated DL models have been employed. Priya et al. (117) examined five different ML approaches (i.e., LASSO, SVM, RF, Ridge, and MLP) to distinguish between 97 GBM and 46 PCNSL cases, with all five approaches sharing similar results. Yet, LASSO had the best performance (0.88 in accuracy and 0.92 in AUC) when using features from the whole tumor, while MLP had the best performance (0.86 in accuracy and 0.91 in AUC) when only using the features from the single largest slice. For an even larger dataset (i.e., 160 GBM and 160 PCNSL), McAvoy et al. (118) applied a CNN variant, EfficientNet (119), and by using TL based on ImageNet, they achieved an accuracy of 0.93 and AUC of 0.94.

#### 4) Differentiating treatment effects versus true disease progression

Pseudoprogression (PsP) is the apparent growth of a lesion or development of new lesions on imaging that represents inflammatory treatment-related changes but looks just like viable tumor growth on MRI. PsP is most common between 3 and 6 months after the completion of radiation therapy, and the corresponding imaging findings will subside on their own over time (120). PsP is more likely in *MGMT* promoter-methylated tumors treated with temozolomide. The increased contrast enhancement on MRI may be caused by the increased vascular permeability from cytotoxic therapies including radiotherapy and chemotherapies such as temozolomide, which may benefit patients receiving immunotherapy and temozolomide but often leads to premature discontinuation of treatment owing to the false judgment of progression of disease (121). Radiation necrosis is another treatment effect that can occur any time after radiation therapy but is most common 1–2 years after radiation. It should be differentiated from true progression of viable tumor before treatment changes are contemplated. As a result, accurate differentiation between treatment effect (i.e., pseudoprogression or radiation necrosis) and true tumor progression is critical in the treatment decision. PsP may be associated with a survival advantage. A key radiology tool in differentiating pseudoprogression or radiation necrosis from true progression of disease is dynamic susceptibility contrast (DSC) MR perfusion-weighted imaging (PWI). Elevated corrected relative cerebral blood volume (crCBV) relative to normal-appearing white matter is more common in a viable tumor than in treatment effects (122). However, PWI is unreliable in patients treated with immunotherapy such as immune checkpoint inhibitors, such that, per immunotherapy response assessment in neuro-oncology (iRANO), the patient is followed for 3 months and then a determination is made of whether the initial increase in size of the lesion represented treatment effects or a viable tumor (123–125).

Booth et al. (126) first analyzed the tumor heterogeneity in T2 MRI using topological descriptors called Minkowski functionals (MFs). Then they utilized an SVM model, together with image features such as MFs, size, and signal intensity, to distinguish between pseudoprogression and true progression, and achieved an accuracy of 0.88, slightly higher than using RF for feature selection and LASSO for classification (0.86). Hu et al. (127) took advantage of T1 MRI and other eight-dimensional feature vectors, including T2, FLAIR, proton density, ADC, PWI, derived relative cerebral blood volume (rCBV), relative cerebral blood flow (rCBF), and mean transit time maps, to train an SVM model, and achieved an AUC of 0.94 in distinguishing between pseudoprogression and true progression. The ADC map derived from DWI and rCBV and rCBF derived from PWI were found to make a greater contribution to the discrimination than the conventional radiology images do.

Due to the time correlation embedded in true progression and PsP radiology data, Lee et al. (128) and Jang et al. (129) exploited recursive LSTM-CNN structures on MRI to distinguish between the two occurrences. In comparison to Lee’s multimodal MRI data (i.e., T1, T2, FLAIR), Jang et al. (129) combined/fused T1 MRI data with clinical features to develop an LSTM-CNN clinic-feature-fused model and achieved an AUC of 0.87 and F1 score of 0.74, outperforming the model trained with MRI data only and the RF-based model.

Akbari et al. (130) employed TL with a CNN pretrained on ImageNet and feature extraction based on four structural MRIs (i.e., T1, T1-ce, T2, FLAIR), diffusion tensor imaging (DTI), and PWI (rCBV, peak height (PH), percentage signal recovery (PSR)) and achieved an accuracy of 0.84 and AUC of 0.83. Ismail et al. (131) extracted 30 global and local shape features from T1-ce, T2, and FLAIR images and used an SVM classifier to achieve an accuracy of 0.90 in distinguishing PsP from true tumor progression.

In addition to the ambiguity between PsP and true tumor progression, immunotherapies in GBM also suffer from the lack of reliable evaluation methods on the radiological imaging manifestation regarding the alteration of the tumor immune microenvironment (TME, e.g., tumor immune cell infiltration, functional characterization of immune effector/suppressive cells, gene expression profile of immunostimulatory/immunosuppressive cells), a crucial parameter for assessment of intratumoral immune responses (5). In their pioneering work (132), Narang et al. utilized T1-weighted post-contrast and T2-FLAIR images in combination with T-cell surface marker CD3D/E/G mRNA expression data from 78 GBM patient-derived TCGA data to extract six imaging features that are associated with intra-GBM CD3 activity. These imaging features were further trained and tested using an internal dataset from 69 GBM patients that has immunohistochemically (IHC) validated intratumoral CD3 counts. The image-based intra-GBM CD3+ T-cell infiltration model reaches an accuracy of 97.1% and AUC of 0.993 for the training set, with an accuracy of 76.5% and AUC of 0.847 in the test group. A similar study has been reported recently in lower-grade gliomas (LGG) with an expansion from CD3 expression data to multiple immune gene expression profiles, including major histocompatibility complex (MHC)-related molecules, immune checkpoint molecules, and effector/suppressor immune cells (94). In this study, radiomic features extracted by a deep learning neural network-based model have been demonstrated to predict the TME-associated signature immunophenotype mRNAs with an AUC of 0.821 in the test group. Unfortunately, there is no IHC validation on expression of signature immune genes in the test group specimens.

To date, although numerous ML models for differentiating PsP from true tumor progression have been proposed and tested, none of them have been prospectively validated, reflecting the lack of confidence in clinicians to apply these radiomic approaches in their clinic practice. Multiple factors can lead to this significant issue, such as difficulty to applying small sample size-derived prediction models to a large population cohort, poor reproducibility, and lack of consistency between various ML models and/or datasets (further discussed in Section 4). One of the important and challenging factors is that currently there is no clear objective histological definition of pseudoprogression. In a representative study by Melguizo-Gavilanes et al. (133), MRI images and surgical resection-derived histological data from 34 patients with GBM were retrospectively reviewed. Only one-third of the cohort (11/34) demonstrated a concordance for PsP between radiological interpretation and histological diagnosis, whereas the majority of the patients had a histologically “mixed” pattern with tumor and treatment effect, indicating that even histology might not be applied as a gold standard to differentiate PsP and tumor true progression.

### Overall survival prediction

Overall survival prediction of GBM patients provides useful information for surgical and treatment planning. Conventional survival prediction based on clinical information is subjective and could be inaccurate. Radiomic analysis, on the other hand, provides a variety of MRI features to predict disease prognosis, thus providing beneficial information for personalized treatment. Nevertheless, manual feature engineering is still time consuming, laborious, and subjective and may not be able to effectively encode other predictive but implicit information hidden in the multimodal neuroimages (134). Thus, an accurate, generalized yet automated OS prediction is desired.

Macyszyn et al. (135) extracted about 60 features from 105 GBM patients to train an SVM-based predictive model for patient survival and molecular subtype. The predictors were evaluated in 29 new patients and achieved a three-way (long/medium/short survival for longer than 18 months, between 6 and 18 months, and shorter than 6 months) accuracy of about 0.80. Another classifier was trained to discriminate among each of the various GBM molecular subtypes and achieved an accuracy of about 0.76. Sanghazni et al. (136) derived texture features (e.g., first-order texture features, GLCM), tumor shape and volumetric features, and patient ages from 173 patients’ multimodal MRI data (e.g., T1-ce, T2, and Flair) and used an SVM-RFE-based ML model to perform binary (i.e., short and long‘s threshold upon 400 days) and multiclass (i.e., <10, 10~15, and >15 months) OS prediction. Prediction accuracies of 0.987 and 0.89 were achieved for binary and multiclass predictions, respectively.

Choi et al. (137) collected 250 radiomic features extracted from 296 LGG cases from institutional and TCGA/TCIA datasets. They trained three random survival forest (RSF, i.e., a variant of RF) models with 1) these radiomic features; 2) non-imaging prognostic factors including age, resection extent, WHO grade, and *IDH* status; and 3) combination of 1 and 2 on the institutional dataset and validation of the model on the TCGA/TCIA dataset. When applying radiomic features or non-imaging features alone, the two RSF models achieved an AUC of 0.620 and 0.627, respectively. When applying radiomic features together with non-imaging prognostic parameters, the AUC was improved to 0.709. Similarly, in a GBM hypoxia-associated radiomic study, Beig et al. (138) also revealed that when combining clinical features (age, gender, and Karnofsky Performance Score (KPS)) with 270 radiomic features, the concordance index for survival prediction rises to 0.83 in comparison to 0.74 when using radiomic features alone (138). Grist et al. (139) examined various analysis techniques on survival predictions through perfusion and MRI data, especially DWI, collected from 69 pediatric patients. Approaches included conventional regressions and Bayesian analysis on apparent diffusion coefficient (ADC) maps, uncorrected and corrected cerebral blood volume (uCBV and cCBV) maps, and K2 maps (140) and achieved an AUC between 0.63 and 0.82. Supervised (i.e., SVM, RF, and a single-layer neural network) and unsupervised (i.e., k-means clustering) ML analyses achieved an accuracy between 0.90 and 0.98 in distinguishing between high- and low-risk clusters, with distinct differences in survival. In addition to the above models, the Tiwari group has developed a radiomic risk score in which the extracted GBM radiomic features were trained by various Cox regression-based algorithms for survival stratification with an overall concordance index at 0.7 to 0.8 (141–143).

Nie et al. (134) proposed a two-stage learning-based method to predict the OS of HGG patients. Specifically, in the first stage, they adopted a CNN to extract implicit features from multiparametric maps that are computed by multimodal multichannel MRI (i.e., T1-ce, DTI, and rs-fMRI) from 68 HGG patients. Then, those radiomic features along with the demographic and tumor-related features (e.g., age, tumor size, and histological type) were trained in an SVM to model OS prediction (i.e., long or short overall survival time, with a threshold of 650 days). The experimental results demonstrated an accuracy of up to 0.91.

### Identifying biomarkers of brain tumors

Radiogenomics uses radiomics techniques to predict the genetic makeup of tumors. This promotes precision medicine by identifying patients with tumor molecular markers that can be targeted by particular drugs and by predicting how aggressive a tumor will behave, with implications for survival and treatment choice. *Via* exploring the implicit correlation between radiological images and genomic data such as DNA microarrays, microRNA, RNA-Seq, ML techniques can help improve the effectiveness and efficiency in identifying the biomarkers of brain tumors (144).

#### Isocitrate dehydrogenase (*IDH*) mutation

Since the initial reworking of the WHO CNS Tumor Classification System in 2016, genetic biomarkers have become increasingly important in the classification of brain tumors. Isocitrate dehydrogenase is an enzyme in the Krebs cycle, and its mutated gene (*IDH*) is an oncogene. The mutant IDH enzyme produces an oncometabolite 2-hydroxyglutarate (2HG) (145), which promotes the growth of various cancers throughout the body. In brain tumors, *IDH*-mutated tumors are less aggressive than *IDH* wild-type tumors, yet they can convert to the latter. In the 2021 WHO CNS Tumor Classification System, only *IDH* wild-type tumors are classified as GBMs. It is of utmost importance for therapeutic planning to differentiate between the *IDH* mutation and *IDH* wild type, and it would greatly benefit patients if this determination could be done non-invasively and obviate biopsy or resection. Yogananda et al. (33) developed a 3D Dense-UNet network using (a) T2 images only (T2-net) and (b) a combination of T1-ce, T2, and FLAIR images (TS-net) from TCIA and TCGA to non-invasively predict *IDH* mutation. The T2-net demonstrated a mean cross-validation accuracy of 0.97 (sensitivity 0.97, specificity 0.98, AUC 0.98), and TS-net demonstrated a mean cross-validation accuracy of 0.97 (sensitivity 0.98, specificity 0.97, AUC 0.99). In addition, this model automatically segmented the tumor to show areas with either *IDH* mutation or *IDH* wild type. Dice scores were 0.85 for T2-net and 0.89 for TS-net. The benefit of being able to use only T2-weighted images is that gadolinium-based contrast material, which deposits in the brain to unknown effect, does not have to be administered and T2-weighted images can be quickly acquired and are less sensitive to motion artifact.

#### *MGMT* promoter methylation

*MGMT* promoter methylation predicts less aggressive glioma behavior for both *IDH*-mutated and *IDH*-wild-type gliomas. When its promoter is methylated, the *MGMT* gene, which is involved in DNA repair, is hindered and the tumor has greater difficulty overcoming the damage caused by chemotherapy such as temozolomide. Yogananda et al. (146) used a 3D-dense UNet on only T2 images to simultaneously segment the tumor and predict the presence of *MGMT* promoter methylation with a mean three-fold cross-validation accuracy of 0.95 (sensitivity 0.96, specificity 0.92, AUC 0.93, Dice score 0.82).

#### *H3K27M* alterations

In 2016, the WHO released a new histological diagnosis in the classification of CNS malignancies: diffuse midline glioma (DMG), *H3K27M*-mutant. It was renamed as *H3K27M*-altered in 2021 because there are multiple mechanisms involved. These WHO grade 4 tumors are found in or near the midline in the brainstem, thalamus, spinal cord, pineal region, hypothalamus, and cerebellum and exhibit aggressive clinical behavior (147, 148). *H3K27M* is the most frequent mutation in brainstem gliomas (BSGs) (149). Su et al. (150) extracted radiomics features from FLAIR images from 40 patients with *H3K27M* mutations and 60 wild-type patients, all with midline gliomas. The Tree-based Pipeline Optimization Tool (TPOT) was applied to optimize the ML pipeline and select important radiomics features. A total of 10 independent TPOT ML models were compared and tested on 22 independent cohorts of patients, achieving an accuracy ranging from 0.6 to 0.84, and the AUC from 0.73 to 0.90. Pan et al. (149) included a total of 151 patients with newly diagnosed BSGs. A total of 1,697 features, including six clinical parameters and 1,691 imaging features (e.g., GLCM, LBP), were extracted from pre- and post-contrast T1 and T2 images. Spearman’s correlation and relief algorithm were applied for feature selection. Thirty-six MRI features and three clinical features remained and were fed to an RF model to predict *H3K27M* mutations. For comparison, a least-square estimation method-based ML model was developed by utilization of the KPS at diagnosis, symptom duration at diagnosis, and edge sharpness on T2, which achieved an accuracy of 0.80 and AUC of 0.79 in the test cohort if using MRI features alone but can be improved to 0.84 and an AUC of 0.83 if integrated with clinical parameters. The simplified model achieved an AUC of 0.78. Zhuo et al. (151) studied 81 BSG patients with APT imaging at 3T MR and known *H3K27M* status. APTw values (i.e., mean, median, and max) and radiomic features within manually delineated 3D tumor masks were extracted. *H3K27M*-mutant prediction using APTw-derived radiomics was conducted using various models, such as SVM, AdaBoost, autoencoder, LASSO regression, and RF, which achieved an accuracy of 0.86 and an AUC of 0.93 as validated by a prospective cohort of 29 BSG patients.

### Discussion

Despite that numerous ML studies have been conducted in GBM radiomic analysis, comparing the results from individual articles is not a trivial task due to the use of different data sets. The accuracies, AUCs, and Dice scores in different studies may vary from 0.7 to 0.98: most state-of-the-art studies using public datasets (e.g., BraTS) achieve an accuracy of 0.84–0.94, but some studies with certain private data can reach 0.98. Meanwhile, current major public datasets also lack sub-categories for brain tumor classification and segmentations, which restricts the development of a more powerful and comprehensive ML-model to distinguish more brain tumor types. Without sufficiently large datasets, ML models with too many parameters (i.e., neurons in each layer and the number of layers) are easily overfitting to a specific dataset, losing the generalizability of the model to other patient groups.

## Challenges and perspectives on future AI/ML techniques

### Overview of current challenges in ML-based radiomic neuro-oncology studies

As ML is a data-driven statistical approach to extract common features within different data samples, sufficient imaging datasets are required to train advanced ML models and to fairly evaluate their performances (e.g., accuracy, Dice score, AUC) in the field of neuro-oncology. Currently, only a limited number of brain tumor sub-categories have been analyzed with ML studies while many other brain tumor/disease types have not, due to the lack of labeled/annotated data for training. Examples include differentiating dysembryoplastic neuroepithelial tumor (DNET), ganglioglioma, pleomorphic xanthoastrocytoma (PXA), and multinodular and vacuolating neuronal tumor (MVNT).

However, establishing standardized radiological imaging datasets or standardizing McMv datasets for extensive and generalized ML-based GBM analysis can be manpower and time consuming because most of these datasets require highly accurate manual labeling/annotation to serve as the “ground truth” for the ML model training and validation. In addition, these datasets should be generalizable for various neuro-oncology analyses and patient groups and should be carefully labeled by various disease categories. Additional information (e.g., survival time, related biotest results, related clinical/medical history) may also be necessary for more sophisticated and comprehensive analyses. This requires a continuous update of the datasets, leading to a significant cost of data management (**Figure 1B**).

Another challenge is that current mathematic mechanisms in the ML model are based on statistics, which means there may not be a “deterministic optimal” algorithm or architecture for an ML model to achieve the “ideal/optimal” outcomes. The initial values of the trainable parameters in ML models and the slight differences in structure may affect the training outcome significantly. Even when using public datasets (e.g., BraTS), similar ML networks may yet achieve varying results (152, 153). Thus, many researchers intend to simply add more layers in CNNs to improve the accuracy, potentially causing extensive yet unnecessary computational complexity during the training process but overlooking the biological connections and meaning behind those data. On the other hand, too much engineering (i.e., strong feature extraction, data restriction/collection) in data preprocessing may also lead to overfitting of the ML network to the training data and lose the generalizability of trained ML models for larger populations with more diversity (**Figure 1B**).

### Promising strategies enhancing performance of AI models in GBM radiomic analysis

Aside from using genuine radiological brain tumor images alone to train ML models, three other trends are gaining popularity to improve the model performance in accuracy, Dice score, AUC, and generalizability. The first trend is to use TL (100, 101), which takes advantage of other larger non-neuro-oncology or even non-medical image datasets to pretrain the ML model. Then by keeping the pretrained parameters in the low-level hidden layers (i.e., closer to the input layer) and fine-tuning the ones in the high-level layers and output layers with brain tumor training image datasets, the pretrained ML model can be adopted for brain tumor analysis. Typical image datasets for pretraining ML models includes ImageNet (102), the modified National Institute of Standards and Technology (MNIST) database (154), and International Symposium on Biomedical Imaging (ISBI) (155). However, if the pretraining dataset is drastically unsimilar to the target dataset, the pretraining effect is limited. Therefore, a standardized radiomic medical dataset with various categories is preferred, benefitting not only neuro-oncology studies but also other medical and biomedical studies.

The second trend is to use GANs to generate synthetic data for augmentation (78, 79, 155). However, as discussed in Section 2.4, this approach itself requires a large set of genuine images to train the discriminator network in the GAN, before it can synthesize accurate-appearing brain tumor images to train other ML models for brain tumor analysis.

The third trend is to fuse multimodal data for a more comprehensive analysis. Examples include multimodal MRI (156, 157), combinations of MRI and PET (54), image genomics (i.e., radiogenomics), and clinical data to study the association between imaging biomarkers and genomic characteristics (144, 149–151, 158, 159). Especially for radiogenomics, some studies (144, 158, 159) have identified associations between quantitative image features and gene expression profiles of glioblastoma (e.g., *H3K27M*, *TP53*, *EGFR*, *NF1*, and *IDH1*) and its molecular subtypes (e.g., classical, mesenchymal, proneural, and neural). Additional studies indicate that quantitative MR imaging features derived from entire tumor volumes can be used to identify glioblastoma subtypes with distinct molecular pathways (160, 161). With the help of additional complementary correlated features from different types of radiomic images and/or genomic information, or simply just the medical history of the patients, ML can take advantage of data to achieve more accurate predictions (**Figure 1B**).

### Outlook on teamwork among computer scientists/engineers, physicians, and biomedical researchers

As aforementioned, high-quality clinical data and labels/annotations are critical to ML algorithms for both accuracy and generalizability, and biological knowledge can help extract certain features to improve the accuracy as well as the training efficiency. Therefore, strong collaborations should be established among computer scientists, engineers, physicians, and biomedical researchers to facilitate the standardization and enrichment of neuro-oncology radiomic datasets and the development of innovative and more advanced AI/ML models (**Figure 1B**). In addition, with larger amounts of data to track patients’ treatment process and the outcomes, it is even possible to develop ML/AI techniques to determine more suitable plans for their treatment, to improve the patients’ survival time as well as their quality of life.

## Conclusion

With the urgent needs for highly accurate and automatic analysis of brain tumors and the rapid growth of clinical imaging data, image-based ML/AI techniques are playing an increasingly important role. Various combinations of feature extraction algorithms and ML models have been implemented and have achieved comparable or even better performance than manual analysis. However, challenges remain for exploring cancer heterogeneity, higher prediction accuracy, and generalizability for larger, more diverse patient groups. We believe that, by improving dataset quality, employing multimodal data fusion, developing more advanced ML models, and further enhancing collaborations between computer scientists, engineers, physicians, and biomedical researchers, AI techniques will accelerate quantitative cancer imaging analysis for clinical applications with great improvements in patient care.

## Author contributions

MZ, YK, VH, LZ, and SZ formulated the conception of this review. MZ, SL, and SZ performed initial screening of the citations, datasets, and related AI/ML techniques. MZ, SL, LZ, and VH wrote and reviewed the manuscript and prepared the figures. AH provided critical comments for the manuscript. All authors approved the manuscript for publication and declare no competing financial interest.

## Funding

This study was supported by the Nevada Governor’s Office of Economic Development (GOED) AWD-02-00001154 to SZ.

## Acknowledgments

Thanks for UNLV University Libraries Open Article Fund to support this open-access journal article.

## Conflict of interest

The authors declare that the research was conducted in the absence of any commercial or financial relationships that could be construed as a potential conflict of interest.

## Publisher’s note

All claims expressed in this article are solely those of the authors and do not necessarily represent those of their affiliated organizations, or those of the publisher, the editors and the reviewers. Any product that may be evaluated in this article, or claim that may be made by its manufacturer, is not guaranteed or endorsed by the publisher.
